# A new crystal form and anti­microbial activity of (*E*)-1-[3-(2-hy­droxy­benzyl­idene­amino)­phen­yl]ethanone

**DOI:** 10.1107/S205698901800806X

**Published:** 2018-06-05

**Authors:** Nabila Moussa Slimane, Zakaria Bouhidel, Aouatef Cherouana

**Affiliations:** a Unité de Recherche de Chimie de l’Environnement et Moléculaire Structurale (URCHEMS), Département de Chimie, Université des freres Mentouri-Constantine 1, 25000 Constantine, Algeria

**Keywords:** crystal structure, Schiff base, new crystal form, Hirshfeld surfaces, anti­microbial activity

## Abstract

The title Schiff base compound, C_15_H_13_NO_2_, crystallizes in a new crystal form in the space group *P*2_1_2_1_2_1_, which is different from the monoclinic *P*2_1_/*n* space group reported previously [De *et al.* (2009 ▸). *Indian J. Chem. Sect. B*, **48**, 595–598]. An intra­molecular O—H⋯N hydrogen bond occurs between the hy­droxy and azomethine moieties. In the crystal, mol­ecules are linked by weak C—H⋯O hydrogen bonds into supra­molecular chains propagating along the *b*-axis direction with a *C*(8) graph-set motif. The contribution of these two contacts in Hirshfeld surface area are around 19 and 21%, respectively. The title compound was screened for its anti­bacterial activity against two gram-negative (*Escherichia coli* and *Salmonella typhimurium*) and one gram-positive (*Staphyloccus aureus)* bacteria. The results of this study reveal that this Schiff base shows good activity against only one bacterium, *i.e. Salmonella typhimurium*.

## Abstract 2

In the ortho­rhom­bic crystal form, the mol­ecules are linked by weak C—H⋯O hydrogen bonds into supra­molecular chains propagating along the *b*-axis direction.

## Chemical context   

Schiff bases (Wang *et al.*, 2008 ▸) are versatile ligands synthesized from the condensation of primary amines with carbonyl groups. These compounds have been shown to exhibit a broad range of biological activities, including anti­fungal, anti­bacterial, anti-malarial, anti­proliferative, anti-inflammatory, anti­viral and anti­pyretic properties (Dhar & Taploo, 1982 ▸; Przybylski *et al.*, 2009 ▸). Imine or azomethine groups are present in various natural, natural-derived and non-natural compounds and have been shown to be critical for their biological activity (Bringmann *et al.*, 2004 ▸; de Souza *et al.*, 2007 ▸).
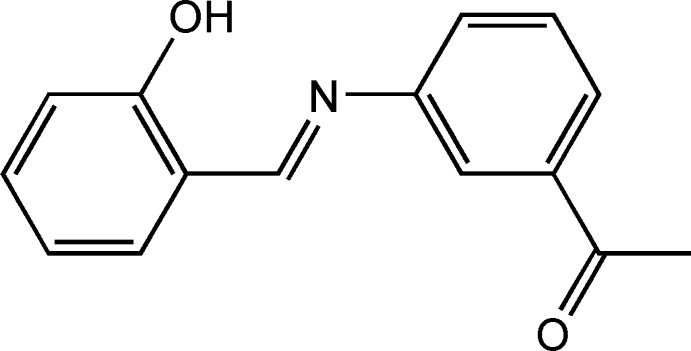


In this paper, we report the structural characterization using X-ray diffraction of the title Schiff base derived from salicyl­aldehyde, including an investigation of the Hirshfeld surfaces and its anti­microbial activity against two gram-negative (*Escherichia coli* and *Salmonella* typhimurium) and one gram-positive (Staphyloccus aureus*)* bacteria. Schiff bases derived from this benzaldehyde are members of one of the most commonly investigated classes of compound, and have attracted the inter­est of chemists and physicists because they show photochromism and thermochromism in the solid state. These photo- and thermochromic features are caused by proton transfer to the N atom from the O atom under the influence of light or temperature, respectively. It has been proposed that mol­ecules showing thermochromism are planar and those showing photochromism are non-planar (Moustakali-Mavridis *et al.*, 1980 ▸; Hadjoudis *et al.*, 1987 ▸).

## Structural commentary   

**Figure 1:**
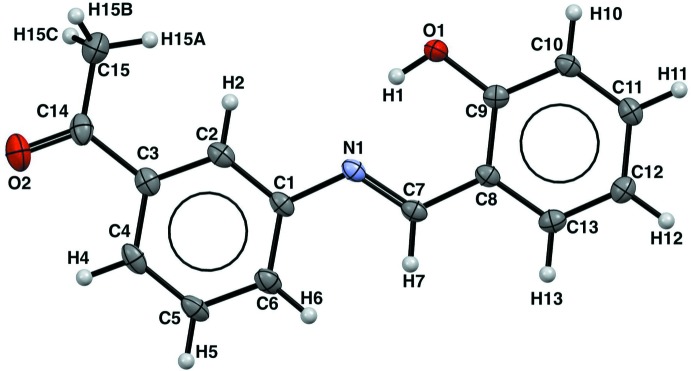
View of the title compound with the atom-numbering scheme. Displacement ellipsoids for non-H atoms are drawn at the 50% probability level.

The title Schiff base (Fig. 1 ▸) consists of two aromatic phenyl rings linked *via* an azomethine (C=N) group. The two phenyl rings are monosubstituted by a hydroxyl group on the same side as the azomethine carbon atom and by an aceto group on the other side. Relevant bond distances and angles are in good agreement with those reported in similar Schiff base compounds (Benarous *et al.*, 2016 ▸; Chen *et al.*, 2011 ▸). The C1—N1—C7—C8 torsion angle of −179.4 (2)° indicates an almost planar *E* configuration with respect to the imine C=N bond, as expected for a compound having an azomethine HC=N bond as this is the most thermodynamically stable configuration (Ciciani *et al.*, 2008 ▸). The N1=C7 [1.293 (3) Å] and C9—C10 [1.394 (3) Å] bond distances indicate that the compound adopts the phenol–imine tautomeric form with an N=C double bond and a C—C single bond (Table 1 ▸). Comparable values are observed in Schiff bases obtained from the same salicyl­aldehyde derivative with phenol–imine tautomeric form (Albayrak *et al.*, 2010 ▸; Şahin *et al.*, 2009 ▸).

An intra­molecular O—H⋯N hydrogen bond (Table 2 ▸) occurs between the O-hydroxyl and N azomethine atoms, forming an *S*(6) ring motif. Such a hydrogen bond is frequently observed in Schiff bases derived from salicyl­aldehyde (Alpaslan *et al.*, 2011 ▸).

## Supra­molecular features   

**Figure 2:**
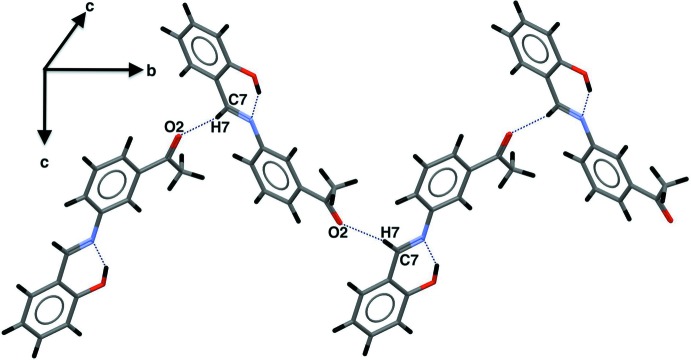
C—H⋯O hydrogen bonds (Table 1 ▸) in the title compound.

**Figure 3:**
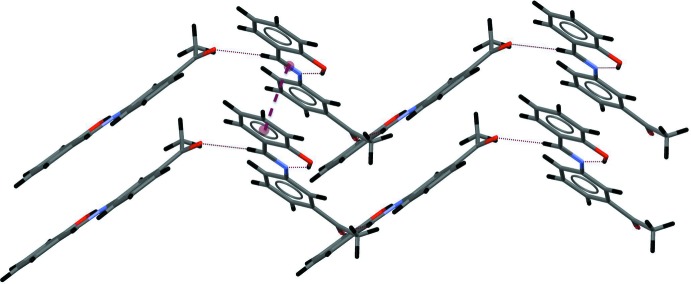
π–π inter­action between a benzene ring and the C=N double bond.

In the crystal, the mol­ecules are linked *via* C—H⋯O hydrogen bonds between the carbon atom of azomethine group and the oxygen atom of the meth­oxy substituent, generating infinite chains with graph-set motif *C*(8) along the *b*-axis direction (Table 2 ▸, Fig. 2 ▸). The chains are linked *via* π–π inter­actions (3.535 Å) between the C8–C13 benzene ring and the C=N double bond (Fig. 3 ▸).

## Hirshfeld surfaces analysis   

**Figure 4:**
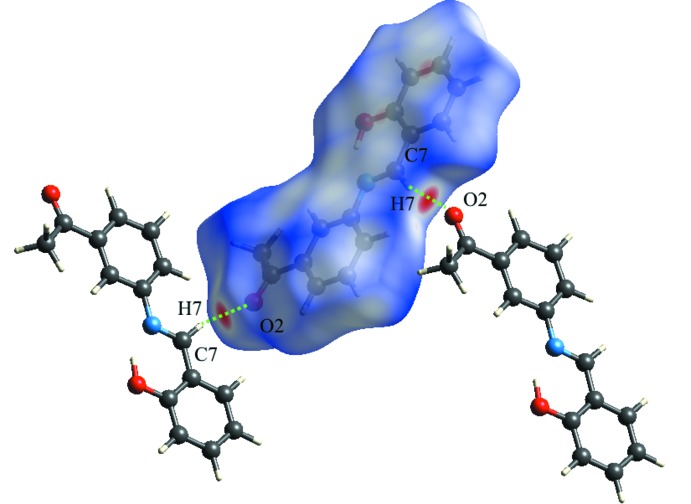
Hirshfeld surface of the title compound mapped over *d*_norm_ (−0.60 to 0.90 a.u.).

**Figure 5:**
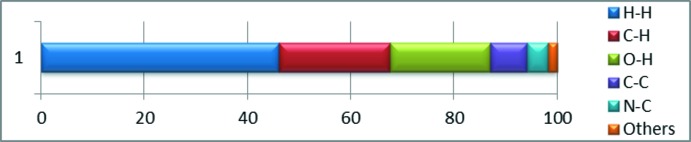
Relative contributions of various inter­actions to the Hirshfeld surface area.

**Figure 6:**
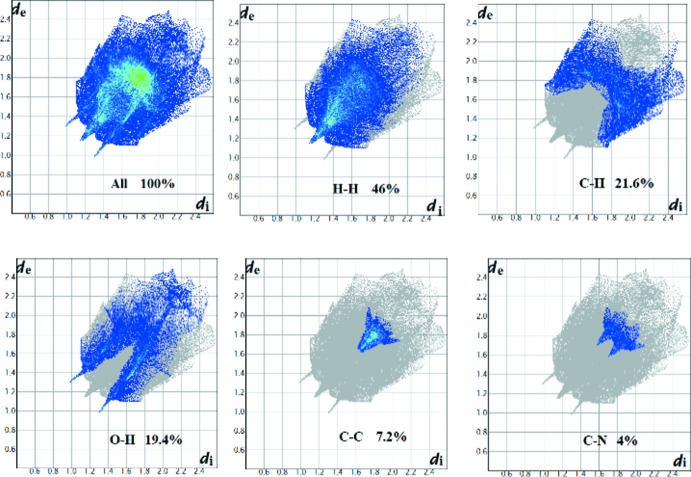
Two-dimensional fingerprints of the compound, showing all inter­actions and H⋯H, C⋯H, O⋯H, C⋯C and C⋯N contacts.

Hirshfeld surfaces and two-dimensional fingerprint plots were generated by *CrystalExplorer* 3.1 (Wolff *et al.*, 2012 ▸) to visualize and explore the inter­molecular inter­actions. These mol­ecular surfaces reflect inter­molecular contacts based on colour coding distances from the surface to the nearest atom exterior (*d*_e_) or inter­ior (*d*_i_) to the surface. In the Hirshfeld surface mapped over *d*_norm_ (Fig. 4 ▸), red indicates the presence of short contacts and white represents contacts around the van der Waals separation, while the blue areas are completely devoid of close contacts. The inter­molecular inter­actions were analysed by a combination of 3D Hirshfeld surfaces and 2D fingerprint plots, showing that the inter­molecular H⋯H contacts make the largest contribution, corresponding to 46% of the total Hirshfeld surface area (Fig. 5 ▸). The presence of short inter­molecular H⋯H contacts is observed in the vicinity of 2.30 Å. These contacts are manifested as white spots on the *d*_norm_ surface and are considered to be weak inter­actions. In the fingerprint plots (Fig. 6 ▸), the C⋯H/H⋯C contacts, representing 21.6% of the total Hirshfeld surface, appear as two short spikes. The red spots on the *d*_norm_ surface in Fig. 4 ▸ are due to the CH⋯O contacts corresponding to the C—H⋯O hydrogen bond. The O⋯H contacts (19.4% of the total Hirshfeld surface) show up as a sharp spike in the fingerprint plots at *d*_e_ + *d*_i_ ≃ 2.3 Å. Finally, the packing cohesion in this structure is also provided by C⋯N and C⋯C inter­actions, which correspond to π–π stacking inter­actions.

## Database survey   

A search for the title compound in the Cambridge Structural Database (Version 2.39; Groom *et al.*, 2016 ▸) revealed that the crystal structure of the title compound had been previously been reported in the monoclinic *P*2_1_/*n* space group (De *et al.*, 2009 ▸). The latter differs from the title structure at position 3 of the aceto substituent, which is on the same side of the hydroxyl group in the title compound and in the opposite side in the reported one. This difference in position directly affects the hydrogen-bonding pattern. Similar infinite C—H⋯O chains occur in both compounds but the angle between linked molecules is *ca* 67.99° in the title compound and 77.61° in the reported one. The CSD search also found seven hits for structures containing the title molecule but with additional substituents (a methoxy or an additional hydroxyl group).

## Anti­microbial activity   

The title compound was screened for its anti­bacterial activity against two gram-negative (*Escherichia coli* and *Salmonella typhimurium*) and one gram-positive (*Staphyloccus aureus)* bacterial strains by the agar-well diffusion method (Cruickshank,1970 ▸). The solvent DMSO was used as negative control. A 0.5 ml spore suspension (10^−6^–10^−7^ spore ml^−1^) of each of the investigated organisms was added to a sterile agar medium just before solidification, then poured into sterile petri dishes (9 cm in diameter) and left to solidify. Using a sterile cork borer (6 mm in diameter), five holes (wells) were made in each dish, and then 5 µL of the tested compound, dissolved in DMSO with different concentrations (C, C/2, C/4, C/8), was poured into these holes. Finally, the dishes were incubated at 310 K for 48 h. Clear or inhibition zones were detected around each hole. DMSO alone (0.5 µL) was used as a control under the same conditions for each organism by subtracting the diameter of inhibition zone resulting with DMSO from that obtained in the study compound. The anti­bacterial activity of Cefotaxime was also measured in comparison to the title compound and used as a standard to reveal the potency of synthesized derivative.

**Figure 7:**
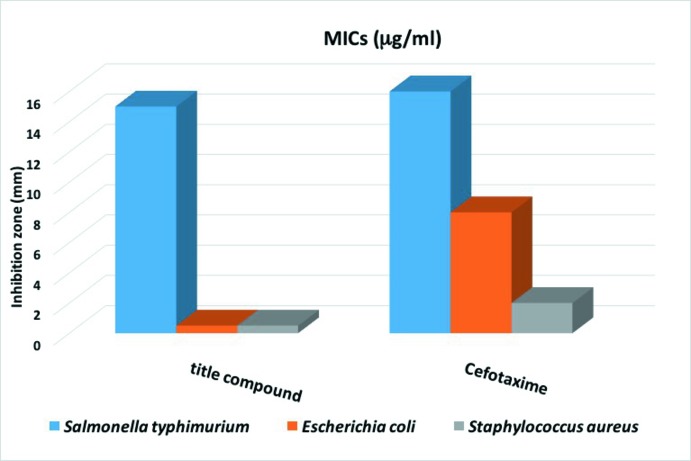
MICs (minimum inhibitory concentrations) anti­bacterial activity of the title compound and the standard Cefotaxime

The results of the anti­microbial screening indicate that the compound shows significant activity only against *Salmonella typhimurium* with an inhibition zone diameter of 15 mm. This value is close to that observed with the standard used (Cefotaxime) against the same bacterium (16 mm). For the two other bacteria, *Escherichia coli* and *Staphyloccus aureus*, the standard exhibits a higher activity than the study compound, for which the inhibition zone diameter is under 2 mm. These results are summarized in Fig. 7 ▸, which gives the MICs (minimum inhibitory concentrations) of the title Schiff base and Cefotaxime.

## Synthesis and crystallization   

The title Schiff base was synthesized by reacting 3-amino­aceto­phenone (0.13 g, 1 mmol) and salicyl­aldehyde (0.12 g, 1 mmol) in ethanol (20 ml). The resulting mixture was refluxed for 3 h. Yellow single crystals suitable for single crystal X-ray diffraction studies were obtained by slow evaporation of the solution.

## Refinement   

Crystal data, data collection and structure refinement details are summarized in Table 3 ▸. All H atoms were located in difference electron-density maps and treated as riding on their parent atoms, with C—H = 0.95–0.98 Å with *U*_iso_(H) = 1.2*U*_eq_(C) or 1.5*U*_eq_(Cmeth­yl) and O—H = 0.84 Å with *U*_iso_(H) = 1.5*U*_eq_(O).

## Supplementary Material

Crystal structure: contains datablock(s) global, I. DOI: 10.1107/S205698901800806X/xu5925sup1.cif

Structure factors: contains datablock(s) I. DOI: 10.1107/S205698901800806X/xu5925Isup2.hkl

CCDC reference: 1846610

Additional supporting information:  crystallographic information; 3D view; checkCIF report

## Figures and Tables

**Table 1:** Selected geometric parameters (Å, °)

O1—C9	1.349 (3)	N1—C1	1.415 (3)
O2—C14	1.213 (3)	N1—C7	1.293 (3)
			
C1—N1—C7	121.25 (18)	O1—C9—C10	119.10 (19)
N1—C1—C2	115.86 (19)	O1—C9—C8	121.3 (2)
N1—C1—C6	125.3 (2)	O2—C14—C15	120.9 (2)
N1—C7—C8	120.8 (2)	O2—C14—C3	120.5 (2)

**Table 2:** Hydrogen-bond geometry (Å, °)

*D*—H⋯*A*	*D*—H	H⋯*A*	*D*⋯*A*	*D*—H⋯*A*
O1—H1⋯N1	0.84	1.84	2.590 (2)	148
C7—H7⋯O2^i^	0.95	2.40	3.321 (3)	162

Symmetry code: (i) 
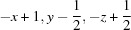
.

**Table 3:** Experimental details

Crystal data	
Chemical formula	C_15_H_13_NO_2_
*M*_r_	239.26
Crystal system, space group	Orthorhombic, *P*2_1_2_1_2_1_
Temperature (K)	100
*a*, *b*, *c* (Å)	4.8637 (3), 14.6601 (10), 16.6512 (9)
*V* (Å^3^)	1187.27 (13)
*Z*	4
Radiation type	Cu *K*α
μ (mm^−1^)	0.72
Crystal size (mm)	0.1 × 0.1 × 0.08

Data collection	
Diffractometer	Oxford Diffraction Xcalibur Sapphire2 CCD
Absorption correction	Integration (*ABSORB*; DeTitta, 1985 ▸)
*T*_min_, *T*_max_	0.966, 0.991
No. of measured, independent and observed [*I* > 2σ(*I*)] reflections	10738, 2461, 2222
*R*_int_	0.058
(sin θ/λ)_max_ (Å^−1^)	0.632

Refinement	
*R*[*F* ^2^ > 2σ(*F* ^2^)], *wR*(*F* ^2^), *S*	0.046, 0.131, 1.07
No. of reflections	2461
No. of parameters	163
H-atom treatment	H-atom parameters constrained
Δρ_max_, Δρ_min_ (e Å^−3^)	0.33, −0.36
Absolute structure	Flack *x* determined using 838 quotients [(*I* ^+^)−(*I* ^−^)]/[(*I* ^+^)+(*I* ^−^)] (Parsons *et al.*, 2013 ▸)
Absolute structure parameter	0.00 (19)

Computer programs: *CrysAlis PRO* (Rigaku OD, 2015 ▸), *SIR2004* (Burla *et al.*, 2005 ▸), *SHELXL* (Sheldrick, 2015 ▸) and *OLEX2* (Dolomanov *et al.*, 2009 ▸).

## supplementary crystallographic information

### Crystal data

**Table:**

C_15_H_13_NO_2_	*F*(000) = 504
*M**_r_* = 239.26	*D*_x_ = 1.339 Mg m^−^^3^
Orthorhombic, *P*2_1_2_1_2_1_	Cu *K*α radiation, λ = 1.54184 Å
Hall symbol: P 2ac 2ab	Cell parameters from 10738 reflections
*a* = 4.8637 (3) Å	θ = 4.0–77.0°
*b* = 14.6601 (10) Å	µ = 0.72 mm^−^^1^
*c* = 16.6512 (9) Å	*T* = 100 K
*V* = 1187.27 (13) Å^3^	Prism, yellow
*Z* = 4	0.1 × 0.1 × 0.08 mm

### Data collection

**Table:**

Oxford Diffraction Xcalibur Sapphire2 CCD diffractometer	2461 independent reflections
Radiation source: fine-focus sealed tube	2222 reflections with *I* > 2σ(*I*)
Graphite monochromator	*R*_int_ = 0.058
φ and ω scans	θ_max_ = 77.0°, θ_min_ = 4.0°
Absorption correction: integration (ABSORB; DeTitta, 1985)	*h* = −5→6
*T*_min_ = 0.966, *T*_max_ = 0.991	*k* = −18→18
10738 measured reflections	*l* = −20→20

### Refinement

**Table:**

Refinement on *F*^2^	H-atom parameters constrained
Least-squares matrix: full	*w* = 1/[σ^2^(*F*_o_^2^) + (0.0633*P*)^2^ + 0.6475*P*] where *P* = (*F*_o_^2^ + 2*F*_c_^2^)/3
*R*[*F*^2^ > 2σ(*F*^2^)] = 0.046	(Δ/σ)_max_ < 0.001
*wR*(*F*^2^) = 0.131	Δρ_max_ = 0.33 e Å^−^^3^
*S* = 1.07	Δρ_min_ = −0.36 e Å^−^^3^
2461 reflections	Absolute structure: Flack *x* determined using 838 quotients [(*I*^+^)-(*I*^-^)]/[(*I*^+^)+(*I*^-^)] (Parsons *et al.*, 2013)
163 parameters	Absolute structure parameter: 0.00 (19)
0 restraints	

### Special details

**Table:**

Geometry. Bond distances, angles etc. have been calculated using the rounded fractional coordinates. All su's are estimated from the variances of the (full) variance-covariance matrix. The cell esds are taken into account in the estimation of distances, angles and torsion angles

### Fractional atomic coordinates and isotropic or equivalent isotropic displacement parameters (Å^2^)

**Table:**

	*x*	*y*	*z*	*U*_iso_*/*U*_eq_	
O1	−0.2016 (4)	0.05562 (11)	0.50631 (9)	0.0218 (4)	
O2	0.9481 (4)	0.29985 (14)	0.27572 (12)	0.0356 (6)	
N1	0.0723 (4)	0.03478 (13)	0.37418 (10)	0.0173 (5)	
C1	0.2784 (5)	0.06549 (15)	0.32063 (12)	0.0175 (6)	
C2	0.4217 (5)	0.14349 (16)	0.34432 (13)	0.0202 (6)	
C3	0.6303 (5)	0.17989 (15)	0.29616 (13)	0.0201 (6)	
C4	0.6939 (5)	0.13896 (17)	0.22293 (13)	0.0225 (6)	
C5	0.5530 (5)	0.06016 (17)	0.19957 (13)	0.0233 (6)	
C6	0.3462 (5)	0.02425 (17)	0.24763 (13)	0.0225 (7)	
C7	−0.0656 (5)	−0.03891 (15)	0.36008 (13)	0.0183 (6)	
C8	−0.2785 (5)	−0.06931 (15)	0.41500 (13)	0.0173 (6)	
C9	−0.3394 (5)	−0.02099 (14)	0.48639 (12)	0.0174 (6)	
C10	−0.5447 (5)	−0.05303 (15)	0.53750 (13)	0.0196 (6)	
C11	−0.6896 (5)	−0.13149 (15)	0.51850 (13)	0.0203 (6)	
C12	−0.6348 (5)	−0.17920 (15)	0.44765 (14)	0.0208 (6)	
C13	−0.4307 (5)	−0.14870 (15)	0.39705 (13)	0.0199 (6)	
C14	0.7915 (5)	0.26259 (16)	0.32198 (15)	0.0243 (7)	
C15	0.7613 (6)	0.29571 (19)	0.40716 (18)	0.0354 (8)	
H1	−0.08597	0.06773	0.47038	0.0326*	
H2	0.37706	0.17221	0.39377	0.0242*	
H4	0.83170	0.16435	0.18922	0.0270*	
H5	0.59906	0.03096	0.15045	0.0280*	
H6	0.25006	−0.02878	0.23072	0.0270*	
H7	−0.02712	−0.07358	0.31321	0.0219*	
H10	−0.58548	−0.02092	0.58561	0.0235*	
H11	−0.82777	−0.15309	0.55406	0.0244*	
H12	−0.73747	−0.23226	0.43451	0.0250*	
H13	−0.39171	−0.18163	0.34925	0.0239*	
H15A	0.62683	0.25763	0.43540	0.0531*	
H15B	0.69811	0.35919	0.40695	0.0531*	
H15C	0.93926	0.29186	0.43454	0.0531*	

### Atomic displacement parameters (Å^2^)

**Table:**

	*U*^11^	*U*^22^	*U*^33^	*U*^12^	*U*^13^	*U*^23^
O1	0.0218 (8)	0.0262 (8)	0.0173 (7)	−0.0043 (6)	0.0034 (6)	−0.0041 (6)
O2	0.0345 (11)	0.0348 (10)	0.0376 (10)	−0.0070 (8)	0.0067 (9)	0.0108 (8)
N1	0.0156 (9)	0.0224 (9)	0.0140 (8)	0.0023 (7)	0.0002 (7)	0.0011 (7)
C1	0.0160 (10)	0.0225 (10)	0.0141 (10)	0.0040 (9)	0.0006 (8)	0.0045 (8)
C2	0.0193 (11)	0.0239 (11)	0.0173 (10)	0.0052 (9)	0.0013 (9)	0.0024 (8)
C3	0.0181 (12)	0.0220 (10)	0.0202 (10)	0.0032 (8)	−0.0003 (8)	0.0057 (8)
C4	0.0174 (11)	0.0333 (12)	0.0169 (10)	0.0035 (10)	0.0011 (9)	0.0103 (9)
C5	0.0212 (12)	0.0349 (12)	0.0139 (9)	0.0035 (10)	0.0016 (9)	0.0015 (9)
C6	0.0182 (12)	0.0337 (12)	0.0155 (10)	−0.0008 (10)	−0.0008 (8)	0.0002 (9)
C7	0.0196 (11)	0.0217 (10)	0.0135 (9)	0.0035 (9)	−0.0005 (9)	0.0011 (8)
C8	0.0173 (11)	0.0206 (10)	0.0141 (10)	0.0021 (8)	−0.0011 (8)	0.0014 (8)
C9	0.0183 (11)	0.0188 (9)	0.0151 (9)	0.0027 (8)	−0.0033 (8)	0.0015 (8)
C10	0.0201 (11)	0.0239 (10)	0.0147 (9)	0.0016 (9)	0.0001 (8)	−0.0002 (8)
C11	0.0175 (10)	0.0242 (10)	0.0193 (10)	0.0014 (9)	0.0014 (9)	0.0043 (8)
C12	0.0207 (12)	0.0183 (10)	0.0235 (11)	−0.0008 (8)	−0.0030 (9)	0.0008 (8)
C13	0.0220 (12)	0.0193 (10)	0.0185 (10)	0.0021 (9)	−0.0034 (9)	−0.0006 (8)
C14	0.0210 (12)	0.0200 (10)	0.0320 (12)	0.0013 (9)	0.0027 (10)	0.0066 (9)
C15	0.0385 (16)	0.0290 (12)	0.0388 (15)	−0.0107 (11)	0.0066 (12)	−0.0038 (11)

### Geometric parameters (Å, º)

**Table:**

O1—C9	1.349 (3)	C10—C11	1.386 (3)
O2—C14	1.213 (3)	C11—C12	1.397 (3)
O1—H1	0.8400	C12—C13	1.377 (3)
N1—C1	1.415 (3)	C14—C15	1.506 (4)
N1—C7	1.293 (3)	C2—H2	0.9500
C1—C6	1.397 (3)	C4—H4	0.9500
C1—C2	1.396 (3)	C5—H5	0.9500
C2—C3	1.399 (3)	C6—H6	0.9500
C3—C4	1.394 (3)	C7—H7	0.9500
C3—C14	1.507 (3)	C10—H10	0.9500
C4—C5	1.398 (3)	C11—H11	0.9500
C5—C6	1.389 (3)	C12—H12	0.9500
C7—C8	1.452 (3)	C13—H13	0.9500
C8—C13	1.411 (3)	C15—H15A	0.9800
C8—C9	1.415 (3)	C15—H15B	0.9800
C9—C10	1.394 (3)	C15—H15C	0.9800
			
O1···N1	2.590 (2)	C5···H11^ix^	2.9900
O2···C7^i^	3.321 (3)	C5···H10^ix^	3.0200
O1···H15B^ii^	2.7200	C6···H10^ix^	2.9800
O1···H5^iii^	2.7600	C6···H7	2.5600
O2···H4	2.5200	C7···H6	2.6500
O2···H6^i^	2.6900	C7···H1	2.4100
O2···H7^i^	2.4000	C9···H5^iii^	2.9700
N1···O1	2.590 (2)	C10···H5^iii^	2.8900
N1···C3^iv^	3.292 (3)	C11···H12^viii^	3.0700
N1···H1	1.8400	C12···H11^viii^	2.8800
C1···C3^iv^	3.594 (3)	C12···H12^viii^	3.0400
C1···C4^iv^	3.448 (3)	C13···H11^viii^	3.0600
C1···C7^v^	3.599 (3)	C15···H2	2.6100
C1···C8^v^	3.320 (3)	H1···N1	1.8400
C1···C9^v^	3.561 (3)	H1···C1	3.0600
C2···C9^v^	3.572 (3)	H1···C7	2.4100
C2···C14^iv^	3.547 (3)	H2···C15	2.6100
C3···N1^v^	3.292 (3)	H2···H15A	1.8800
C3···C1^v^	3.594 (3)	H4···O2	2.5200
C4···C1^v^	3.448 (3)	H4···H12^x^	2.6000
C5···C7^v^	3.563 (3)	H5···O1^xi^	2.7600
C6···C7^v^	3.542 (3)	H5···C9^xi^	2.9700
C7···C6^iv^	3.542 (3)	H5···C10^xi^	2.8900
C7···C5^iv^	3.563 (3)	H6···C7	2.6500
C7···C11^v^	3.485 (3)	H6···H7	2.0300
C7···C13^v^	3.536 (3)	H6···O2^vi^	2.6900
C7···C12^v^	3.278 (3)	H7···C6	2.5600
C7···O2^vi^	3.321 (3)	H7···H6	2.0300
C7···C1^iv^	3.599 (3)	H7···H13	2.4500
C8···C11^v^	3.465 (3)	H7···O2^vi^	2.4000
C8···C1^iv^	3.320 (3)	H10···C5^xii^	3.0200
C8···C12^v^	3.563 (3)	H10···C6^xii^	2.9800
C9···C2^iv^	3.572 (3)	H10···H15B^xiii^	2.6000
C9···C11^v^	3.591 (3)	H11···C5^xii^	2.9900
C9···C1^iv^	3.561 (3)	H11···C12^vii^	2.8800
C11···C8^iv^	3.465 (3)	H11···C13^vii^	3.0600
C11···C7^iv^	3.485 (3)	H12···C11^vii^	3.0700
C11···C12^vii^	3.565 (3)	H12···C12^vii^	3.0400
C11···C9^iv^	3.591 (3)	H12···H4^xiv^	2.6000
C12···C11^viii^	3.565 (3)	H13···H7	2.4500
C12···C7^iv^	3.278 (3)	H15A···C2	2.4700
C12···C8^iv^	3.563 (3)	H15A···H2	1.8800
C13···C7^iv^	3.536 (3)	H15A···H15C^ii^	2.4600
C14···C2^v^	3.547 (3)	H15B···O1^xv^	2.7200
C1···H1	3.0600	H15B···H10^xvi^	2.6000
C2···H15A	2.4700	H15C···H15A^xv^	2.4600
			
C9—O1—H1	109.00	O2—C14—C3	120.5 (2)
C1—N1—C7	121.25 (18)	C1—C2—H2	120.00
N1—C1—C2	115.86 (19)	C3—C2—H2	120.00
N1—C1—C6	125.3 (2)	C3—C4—H4	120.00
C2—C1—C6	118.9 (2)	C5—C4—H4	120.00
C1—C2—C3	120.8 (2)	C4—C5—H5	120.00
C2—C3—C4	119.9 (2)	C6—C5—H5	120.00
C2—C3—C14	121.4 (2)	C1—C6—H6	120.00
C4—C3—C14	118.7 (2)	C5—C6—H6	120.00
C3—C4—C5	119.4 (2)	N1—C7—H7	120.00
C4—C5—C6	120.5 (2)	C8—C7—H7	120.00
C1—C6—C5	120.5 (2)	C9—C10—H10	120.00
N1—C7—C8	120.8 (2)	C11—C10—H10	120.00
C7—C8—C13	119.6 (2)	C10—C11—H11	120.00
C9—C8—C13	118.7 (2)	C12—C11—H11	120.00
C7—C8—C9	121.7 (2)	C11—C12—H12	120.00
O1—C9—C10	119.10 (19)	C13—C12—H12	120.00
C8—C9—C10	119.6 (2)	C8—C13—H13	119.00
O1—C9—C8	121.3 (2)	C12—C13—H13	119.00
C9—C10—C11	120.3 (2)	C14—C15—H15A	109.00
C10—C11—C12	120.8 (2)	C14—C15—H15B	109.00
C11—C12—C13	119.5 (2)	C14—C15—H15C	109.00
C8—C13—C12	121.1 (2)	H15A—C15—H15B	109.00
O2—C14—C15	120.9 (2)	H15A—C15—H15C	109.00
C3—C14—C15	118.5 (2)	H15B—C15—H15C	109.00
			
C7—N1—C1—C2	−177.1 (2)	C3—C4—C5—C6	1.8 (4)
C7—N1—C1—C6	3.0 (3)	C4—C5—C6—C1	−1.0 (4)
C1—N1—C7—C8	−179.4 (2)	N1—C7—C8—C9	−1.3 (3)
N1—C1—C2—C3	179.9 (2)	N1—C7—C8—C13	178.4 (2)
C6—C1—C2—C3	−0.2 (3)	C7—C8—C9—O1	0.1 (3)
N1—C1—C6—C5	−179.9 (2)	C7—C8—C9—C10	−179.7 (2)
C2—C1—C6—C5	0.1 (4)	C13—C8—C9—O1	−179.6 (2)
C1—C2—C3—C4	1.0 (4)	C13—C8—C9—C10	0.7 (3)
C1—C2—C3—C14	−178.4 (2)	C7—C8—C13—C12	−179.7 (2)
C2—C3—C4—C5	−1.8 (3)	C9—C8—C13—C12	−0.1 (3)
C14—C3—C4—C5	177.6 (2)	O1—C9—C10—C11	180.0 (2)
C2—C3—C14—O2	−171.1 (2)	C8—C9—C10—C11	−0.3 (3)
C2—C3—C14—C15	11.1 (3)	C9—C10—C11—C12	−0.7 (3)
C4—C3—C14—O2	9.5 (4)	C10—C11—C12—C13	1.3 (4)
C4—C3—C14—C15	−168.4 (2)	C11—C12—C13—C8	−0.9 (3)

Symmetry codes: (i) −*x*+1, *y*+1/2, −*z*+1/2; (ii) *x*−1/2, −*y*+1/2, −*z*+1; (iii) −*x*+1/2, −*y*, *z*+1/2; (iv) *x*−1, *y*, *z*; (v) *x*+1, *y*, *z*; (vi) −*x*+1, *y*−1/2, −*z*+1/2; (vii) *x*−1/2, −*y*−1/2, −*z*+1; (viii) *x*+1/2, −*y*−1/2, −*z*+1; (ix) −*x*−1/2, −*y*, *z*−1/2; (x) −*x*, *y*+1/2, −*z*+1/2; (xi) −*x*+1/2, −*y*, *z*−1/2; (xii) −*x*−1/2, −*y*, *z*+1/2; (xiii) *x*−3/2, −*y*+1/2, −*z*+1; (xiv) −*x*, *y*−1/2, −*z*+1/2; (xv) *x*+1/2, −*y*+1/2, −*z*+1; (xvi) *x*+3/2, −*y*+1/2, −*z*+1.

### Hydrogen-bond geometry (Å, º)

**Table:**

*D*—H···*A*	*D*—H	H···*A*	*D*···*A*	*D*—H···*A*
O1—H1···N1	0.84	1.84	2.590 (2)	148
C7—H7···O2^vi^	0.95	2.40	3.321 (3)	162

Symmetry code: (vi) −*x*+1, *y*−1/2, −*z*+1/2.
